# Emotion‐related impulsivity factors and intolerance of uncertainty are uniquely associated with interpersonal‐psychological risk factors for suicide

**DOI:** 10.1111/bjc.12535

**Published:** 2025-02-27

**Authors:** Amelia S. Dev, Theresa Davison, Hannah C. Broos, Sheri L. Johnson, Kiara R. Timpano

**Affiliations:** ^1^ University of Miami Coral Gables Florida USA; ^2^ University of California Berkeley California USA

**Keywords:** emotion‐related impulsivity, interpersonal‐psychological theory, intolerance of uncertainty, risk factors for suicide

## Abstract

**Objectives:**

The interpersonal‐psychological theory of suicide identifies three risk factors for suicidal behaviours: perceived burdensomeness, thwarted belongingness, and acquired capability. We sought to clarify relationships between the interpersonal‐psychological risk factors and two individual difference factors, emotion‐related impulsivity (ERI) and intolerance of uncertainty (IU).

**Methods:**

In the current study, we analysed self‐report scales from a large community sample (*N* = 169) that was oversampled for individuals with elevated suicidality. We considered two separate factors of ERI: Pervasive Influence of Feelings, which measures how much a person's emotions shape their worldview, and Feelings Trigger Action, which measures impulsive behavioural reactivity to emotions. We tested unique effects of ERI and IU and the interactions of ERI × IU on the three interpersonal‐psychological risk factors using linear regression models.

**Results:**

Pervasive Influence of Feelings correlated with higher perceived burdensomeness and thwarted belongingness, whereas Feelings Trigger Action correlated with higher perceived burdensomeness and acquired capability. Within the regression models, IU correlated significantly with lower acquired capability but not perceived burdensomeness or thwarted belongingness. Interactions of ERI × IU were not significant.

**Conclusions:**

These results demonstrate the importance of considering both factors of ERI in understanding the risk for suicide. Our results also provide novel evidence that while IU may not impact risk factors such as perceived burdensomeness and thwarted belongingness, higher levels of IU may serve as some protection against individuals' acquired capability for suicidal action. Limitations and implications of findings are discussed.

**Practitioner points**

- There are links between interpersonal‐psychological theory risk factors for suicide and the emotion‐related impulsivity (ERI) constructs of Feelings Trigger Action (impulsive behavioural reactivity to emotions) and Pervasive Influence of Feelings (tendency for someone's emotions to reflexively shape their worldview and diminish motivation). Practitioners should assess patient's levels of these two constructs when attempting to understand a patient's risk for suicide.
- Given other studies have shown Pervasive Influence of Feelings and Feelings Trigger Action to respond to intervention, specifically targeting these ERI factors may help lower risk related to interpersonal–psychological theory risk factors for suicide.
- Our findings suggest a negative association between intolerance of uncertainty (IU) and dangerous or harmful behaviour that contributes to capability for suicide. But because other studies find that higher IU is linked with worse mental health outcomes, it should not be considered a protective factor in general. Practitioners may want to distinguish between low and high IU individuals and assess how their relationship to uncertainty contributes to, or protects against, capability for suicide.

## INTRODUCTION

Suicide is a serious public health concern, with cascading ramifications for the loved ones of those who die by suicide (Jordan, 2008) and for the global mental healthcare system (Shepard et al., 2016). Suicide is among the leading causes of death worldwide, with recent estimates that more than 700,000 people die by suicide every year globally (World Health Organization, 2021). For each adult who dies by suicide, more than 20 others will attempt suicide (World Health Organization, 2014). A growing body of research has sought to identify risk factors for suicidal behaviour, with the aim to improve prevention efforts. Gaining a better understanding of what might give rise to these risk factors, in and of themselves, is an additional important research focus in overarching efforts to reduce suicide and associated sequelae.

One influential theory is the interpersonal‐psychological theory of suicide (Joiner, 2005), which posits a number of risk factors that independently and interactively predict a person's trajectory toward suicidal desire and behaviours (Chu et al., 2017). These factors include perceived burdensomeness, thwarted belongingness, hopelessness, and capability for suicide. Perceived burdensomeness involves a person's sense that they are a liability to the world or others around them, whereas thwarted belongingness involves a person's sense that they cannot make meaningful connections with those around them. Hopelessness refers to a person's sentiment that perceived burdensomeness and thwarted belongingness are immutable. Capability for suicide represents a person's enhanced capacity for suicidal behaviour due to desensitization to the fear of death. The interpersonal‐psychological theory operates within an ideation‐to‐action framework to posit factors influencing suicide ideation and suicide attempts (Klonsky & May, 2014). According to this framework, suicidal desire arises as a result of feeling that burdensomeness and thwarted belongingness are intractable or hopeless. The presence of capability for suicide is theorized to subsequently interact with suicidal desire to further crystallize the trajectory and give rise to suicidal behaviour. While a recent meta‐analysis indicated that the effects of the interpersonal‐psychological factors are generally weak to moderate (Chu et al., 2017), the literature as a whole points to the critical role these risk factors play as valid predictors of suicidal behaviour (e.g., Joiner et al., 2009; Van Orden et al., 2008).

Looking further upstream at cognitive and behavioural individual difference variables that may give rise to or exacerbate the interpersonal–psychological risk factors can help shed light on earlier precipitants of suicide risk, and thus signpost promising directions for future research in this area. Of particular interest are the interpersonal–psychological risk factors of perceived burdensomeness, thwarted belongingness, and capability for suicide, as these represent more immediate risk factors that pertain to cognition and behaviour, and these factors set the stage for downstream components of the interpersonal–psychological theory (i.e. suicide desire, suicide attempts). One construct that may relate to these core risk factors is emotion‐related impulsivity (ERI). ERI is defined as the tendency to react impulsively when experiencing heightened emotions (Whiteside & Lynam, 2001). While several studies have established links between *general* trait impulsivity (i.e., non‐emotional impulsivity) and interpersonal–psychological theory risk factors (e.g., Anestis et al., 2014; Bender et al., 2011), only a handful of studies have examined the relationship between ERI specifically and these risk factors. The majority of past studies measure ERI using the Negative Urgency scale, which assesses tendencies toward rash speech and behaviour during states of high negative emotion. Although these studies support that higher Negative Urgency does correlate with the interpersonal–psychological theory risk factors (Anestis et al., 2011; Anestis & Joiner, 2011), work over the past 10 years has validated a broader conceptualization of ERI that encompasses two separable factors (Carver et al., 2011): Feelings Trigger Action, which reflects impulsive behavioural reactivity to emotions, and Pervasive Influence of Feelings, which captures the cognitive and motivational tendency for someone's emotions to reflexively shape their worldview and for negative emotions to diminish motivation. Both factors of ERI are associated with a broad range of both internalizing (e.g. mood and anxiety) and externalizing symptoms (e.g. anger, hostility, and aggression), although Feelings Trigger Action is more robustly predictive of externalizing symptoms than Pervasive Influence of Feelings (Johnson, Carver, & Joormann, 2013). Furthermore, both ERI factors are associated with suicidal ideation and engagement in nonsuicidal self‐injury, with effects that persist when controlling for other risk factors for suicidal ideation (Johnson et al., 2022).

Findings across several samples therefore indicate that the two ERI factors of Pervasive Influence of Feelings and Feelings Trigger Action can shape suicidal ideation and behaviour, but there is a dearth of work establishing how these ERI factors may differentially relate to risk factors from the interpersonal–psychological theory. Specifically, there is a need to elaborate on evidence suggesting that these ERI factors may show unique associations across the more cognitive and more behavioural interpersonal–psychological theory risk factors. In an adolescent inpatient sample in which the two ERI factors were considered conjointly (Auerbach et al., 2017), Pervasive Influence of Feelings was specifically associated with suicidal ideation and Feelings Trigger Action was associated with past month suicide attempts (Auerbach et al., 2017). In two other samples in which the two forms of ERI were considered conjointly, Pervasive Influence of Feelings was uniquely correlated with a broad array of indicators related to suicidal ideation, including severity of ideation and uncontrollability (Anvar et al., 2022). In line with the ideation‐to‐action framework of the interpersonal–psychological theory, past work has shown thwarted belongingness and perceived burdensomeness as well‐validated indicators of ideation, whereas capability for suicide is more relevant to suicidal behaviour (Joiner et al., 2009). This thought (ideation) versus behaviour (action) distinction may also apply to relationships between the ERI factors and interpersonal–psychological theory risk factors. In line with this, we propose and seek to test whether Pervasive Influence of Feelings, which reflects more internalized cognitive and motivational over‐reactivity to emotions, may be more uniquely related to thwarted belongingness and perceived burdensomeness, whereas Feelings Trigger Action, which reflects behavioural reactivity to emotions, may be more strongly linked with capability for suicide. Clarifying these relationships can help characterize precipitants of risk factors for suicide and thus contribute to research on preventative efforts against suicidal ideation and behaviour.

Another limitation of previous work is that the extant literature has not yet explored additional cognitive constructs that could modulate the relationship between ERI and the interpersonal‐psychological theory factors. One factor that may interact with ERI is intolerance of uncertainty (IU), defined as a person's affective and behavioural response to uncertainty (Carleton, 2016b; Dugas et al., 2004). IU is a transdiagnostic risk factor for multiple mental health indices (e.g., Boswell et al., 2013; Shapiro et al., 2020), including suicidal ideation and cognition (Ciarrochi et al., 2005). Whereas evidence supports the link between IU and suicidal outcomes, only one study to date has explored the relationship between IU and interpersonal‐psychological theory factors. Results from this study suggested that IU was significantly associated with perceived burdensomeness but not with thwarted belongingness (Martin et al., 2021). As IU and ERI have been found to interact in relation to other psychopathology outcomes (Xu et al., 2024), one possibility is that IU could moderate the relationship between ERI and the interpersonal‐psychological theory risk factors. In line with this, researchers have posited that IU may lead to increased suicide risk by exacerbating emotional distress (Allan et al., 2023). We propose that higher emotional distress related to IU could amplify the effect of ERI on suicide risk factors, such that individuals with high levels of both ERI and IU may be at the greatest risk for suicide. To our knowledge, no study has tested the potential synergistic effects of IU and ERI on interpersonal‐psychological theory factors, thus limiting our understanding of how these constructs may work together to predict suicide risk.

In sum, we sought to fill gaps in the literature on ERI, IU, and three factors from the interpersonal‐psychological theory (capability for suicide, perceived burdensomeness, and thwarted belongingness). The study had two primary aims: (1) to examine the unique effects of behavioural (Feelings Trigger Action) and cognitive‐motivational (Pervasive Influence of Feelings) ERI factors and IU on the interpersonal‐psychological factors, and (2) to test whether ERI factors and IU interact in relation to the interpersonal‐psychological factors. We hypothesized that greater Feelings Trigger Action would predict higher capability for suicide, whereas greater Pervasive Influence of Feelings would predict higher perceived burdensomeness and thwarted belongingness, controlling for IU. We also hypothesized that higher IU would correspond with the three interpersonal‐psychological theory factors (capability for suicide, perceived burdensomeness, and thwarted belongingness), controlling for ERI factors. With respect to the synergistic role of IU and ERI, we hypothesized that there would be a significant interaction between IU and each of the ERI factors, such that higher IU would exacerbate the effect of ERI on interpersonal‐psychological factors.

## METHOD

Data was collected as part of a larger investigation that examined the mechanistic underpinnings of ERI in a general clinical community sample (R01MH110477). A supplement to the parent investigation included specific hypotheses related to the role of ERI in suicidal ideation and behaviours (R01 110477S2 Supplement). The current study fell within the scope of that supplement. Procedures specific to the present report are summarized below. A more detailed description of the methods of the parent investigation can be found in Johnson et al. (2022), though none of the present aims or hypotheses were addressed in the previously published report.

### Participants and procedures

Participants were recruited through Amazon Mechanical Turk (MTurk). We restricted the sample to English‐speaking adults over 18 who resided in the United States. To select a sample of individuals with a lifetime history of suicidal ideation, participants completed a prescreening survey and were only invited to complete this study if they endorsed lifetime suicidal ideation. Out of 276 participants who were eligible after prescreening and completed the survey, participants were excluded if they had duplicate MTurk IDs, for careless responding (e.g. failing attention items, selecting the same response for all items in a scale; Meade & Craig, 2012), for completing the survey in an implausibly short amount of time (<10 min), or if they had missing data for the interpersonal‐psychological theory. This resulted in a final sample size of 169. Additional details on exclusion criteria and recruitment procedures can be found in Johnson et al. (2022) [see Sample 2 description].

The final sample was 51.5% female, with a mean age of 37 (*SD* = 11.8, Range = 19–71). The racial distribution of the sample was 78.7% European/Caucasian, 5.9% Asian, .6% American Indian, 10.1% African American, 3% Mixed, and 1.8% other. Approximately 6% of participants identified as Hispanic/Latino.

Data were collected online using the Qualtrics platform (Qualtrics, 2021). Participants provided informed consent and then completed survey measures and questions concerning demographic information, including their age, gender, race, and ethnicity. Participants were paid at a rate of $7 per hour.

### Measures

#### Three factor impulsivity scale (TFII; Carver et al., 2011)

The TFII is a 54‐item self‐report measure of trait impulsivity that includes the Pervasive Influence of Feelings and Feelings Trigger Action subscales. Pervasive Influence of Feelings captures poor constraint over cognitions and motivation in response to mostly negative emotions (e.g., “*A single failure can change me from feeling OK to seeing only the bad in myself*” *and “I respond to feeling sad by just stopping moving”*). Feelings Trigger Action covers tendencies to respond to positive or negative emotions with impulsive speech or behaviours (e.g., *“When I am upset I often act without thinking”*). These factors have been shown as valid across multiple indices of psychopathology, aggression, and suicidality (Auerbach et al., 2017; Johnson et al., 2017; Johnson, Carver, Mulé, & Joormann, 2013). Participants rate each item on a five‐point Likert scale ranging from 1 (*strongly disagree*) to 5 (*strongly agree*). Both the Pervasive Influence of Feelings factor and Feelings Trigger action factor are comprised of three subscales, the contents of which are outside the scope of the present investigation. In brief, these subscales are averaged, and total scores for Pervasive Influence of Feelings and Feelings Trigger Action are calculated by taking the average of these subscale averages such that higher values indicate higher levels of Pervasive Influence of Feelings or Feelings Trigger Action, respectively. For more detail on subscales, see Anvar et al. (2022).

#### Intolerance of uncertainty scale‐short (IUS‐12; Carleton et al., 2007)

The IUS‐12 is a 12‐item measure of emotional and behavioural responses to uncertainty, including items such as *“One should always look ahead so as to avoid surprises”* and “*A small, unforeseen event can spoil everything, even with the best of planning*.” The shortened IUS‐12 retains the internal consistency of the original 27‐item scale, has excellent reliability (*α* = .91), and has been cross‐validated with related measures (Carleton et al., 2007). Participants rate statements on a five‐point Likert scale from 1 (*not at all characteristic of me*) to 5 (*entirely characteristic of me*). Items are summed and higher scores represent higher IU.

#### Interpersonal needs questionnaire (INQ‐15; Van Orden et al., 2008)

The INQ‐15 is a 15‐item self‐report scale measuring Perceived Burdensomeness (six items) and Thwarted Belongingness (nine items), as outlined in the interpersonal‐psychological theory (Joiner, 2005). Perceived burdensomeness encompasses a person's perception that they are a liability on the world and on those around them (e.g., “*These days, the people in my life would be better off if I were gone*”). Thwarted belongingness reflects an absence of meaningful connections and societal acceptance (e.g., *“These days, I often feel like an outsider in social gatherings”*). The INQ demonstrates good psychometrics, including reliability and convergent validity, and shows prospective associations with suicidal ideation (Van Orden et al., 2012). Items are rated on a seven‐point Likert scale ranging from 1 (*not at all true for me*) to 7 (*very true for me*). Scores are summed and higher scores indicate higher levels of perceived burdensomeness and thwarted belongingness.

#### Acquired capability for suicide scale (ACSS; Van Orden et al., 2008)

The ACSS is a 20‐item self‐report questionnaire that assesses the degree to which participants are habituated to the prospect of death (e.g., *“I am not at all afraid to die”*). Psychometrics of the ACSS were originally tested in an investigation of the relationship between impulsivity and capability for suicide, and in this study the ACSS was shown to have adequate reliability, convergent validity, and discriminant validity (Bender et al., 2011). Further work has supported the convergent validity of the ACSS with constructs such as exposure to painful and provocative events (Bryan et al., 2013) and suicide attempt history (Anestis & Joiner, 2011). Participants rate these items on a five‐point Likert scale ranging from 0 (*not at all like me*) to 4 (*very much like me*). Items are summed to calculate a total score. Higher scores indicate greater capability for suicide, or a reduced sense of fear in response to thoughts of death or potentially fatal experiences.

### Data analytic plan

Data were examined for violations of assumptions of normality. All variables had relatively normal distributions (i.e., skewness <3 and kurtosis <8). Pearson correlations were used to explore basic associations between study variables. For our main study aims, we conducted linear regression models to test the independent and interactive effects of our predictors on each interpersonal–psychological theory risk factor. To test Aim 1, we conducted three separate models for each dependent variable (capability for suicide, perceived burdensomeness, and thwarted belongingness). In each model, the respective interpersonal–psychological theory risk factor was regressed on Pervasive Influence of Feelings, Feelings Trigger Action, and IU. To test Aim 2, we added the interaction between Pervasive Influence of Feelings and IU, and the interaction between Feelings Trigger Action and IU. Variables were centered prior to calculating the interaction terms. All analyses were conducted in SPSS Statistical Software version 26 (IBM Corp, 2019) and R statistical software (R Core Team, 2020).

#### Exploratory analyses

Below, we also present exploratory follow‐up analyses for the models testing the effect of ERI factors on capability for suicide. Specifically, we present analyses utilizing a modified 7‐item version of the ACSS that includes only items assessing fear about death (ACSS‐FAD; Ribeiro et al., 2014), and a 13‐item version of the ACSS that omits items pertaining to fear about death and captures pain tolerance and reactions to painful and provocative events (ACSS no‐FAD).

Ribeiro et al. (2014) proposed the single‐factor, 7‐item ACSS‐FAD in response to concerns about a lack of empirical work validating the factor structure of the full 20‐item ACSS. This measure, which includes only the 7 ACSS items assessing fear about death, was based on expert review of the ACSS and subsequent removal of items related to painful and provocative experiences, as these “are causal and not defining aspects of the acquired capability” (i.e. they might *precede* the development of capability for suicide rather than representing a *subfactor of* capability for suicide). The single pain tolerance item from the ACSS was also removed, as including only one item for pain tolerance would not allow for latent measurement of the pain tolerance domain. The 7‐item ACSS‐FAD derived by expert review was validated by confirmatory factor analyses.

Although Ribeiro et al. (2014) suggest the exclusion of items related to painful and provocative experiences, we were specifically interested in testing broad relationships between impulsivity and the full 20‐item ACSS (i.e. including items assessing pain tolerance and painful and provocative experiences), based on theoretical links between impulsivity and the tendency to engage in painful and provocative events (Van Orden et al., 2010). We thus utilized the full 20‐item ACSS for our main analyses as reported above in our a priori Data Analytic Plan, but conducted follow‐up exploratory analyses that replaced the full 20‐item ACSS with the ACSS‐FAD (7‐items) and the ACSS no‐FAD (13‐items). Beyond allowing us to more granularly test relationships between ERI and the non‐FAD ACSS items measuring elevated pain tolerance and reactions to painful and provocative events, this also enabled us to probe for differences in findings across the 7‐item ACSS‐FAD model and the 13‐item version of the ACSS without FAD items.

## RESULTS

Descriptive statistics are shown in Table 1.

**TABLE 1:** Descriptive statistics for key variables (*N* = 169).

	*M*	*SD*	Min	Max	Skew	Kurtosis	*α*
Capability for Suicide (ACSS)	33.36	13.97	2	71	0.15	−0.10	.87
Thwarted Belongingness (INQ)	28.23	13.83	9	63	0.43	−0.65	.94
Perceived Burdensomeness (INQ)	12.53	9.56	6	42	1.37	0.68	.97
Feelings Trigger Action (TFII)	2.33	0.91	1	4.44	0.36	−0.93	.96
Pervasive Influence of Feelings (TFII)	2.89	1.12	1	5	0.06	−1.04	.90
Intolerance of Uncertainty (IUS‐12)	35.97	10.96	14	60	0.00	−0.69	.93

*Note*: Measures used for each variable are added in parentheticals for additional clarity.

Abbreviations: ACSS, Acquired Capability for Suicide Scale; INQ, Interpersonal Needs Questionnaire; IUS‐12, Intolerance of Uncertainty Scale‐Short; TFII, Three Factor Impulsivity Scale.

As background for regression models, Pearson's correlations among study measures are shown in Table 2. As expected, there were significant positive correlations between the interpersonal‐psychological theory risk factors. In addition, the ERI factors were positively correlated with each other and with IU. Both ERI factors and IU showed strong positive relationships with thwarted belongingness and with perceived burdensomeness; however, there were no significant associations with capability for suicide. Due to the presence of significant positive correlations between our key variables, we assessed for multicollinearity values between our planned predictors for regression models (Feelings Trigger Action, Pervasive Influence of Feelings, and IU by measuring the variable inflation factor (VIF)). The VIF was 1.51 for Feelings Trigger Action, 2.09 for Pervasive Influence of Feelings, and 1.66 for IU.

**TABLE 2:** Correlations between key variables.

	1	2	3	4	5
Capability for Suicide (ACSS)					
2 Thwarted Belongingness (INQ)	0.19*				
3 Perceived Burdensomeness (INQ)	0.26***	0.59***			
4 Feelings Trigger Action (TFII)	0.14	0.39***	0.51***		
5 Pervasive Influence of Feelings (TFII)	0.02	0.55***	0.52***	0.58***	
6 Intolerance of Uncertainty (IUS‐12)	−0.11	0.41***	0.35***	0.41***	0.63***

*Note*: Measures used for each variable are added in parentheticals for additional clarity.

Abbreviations: ACSS, Acquired Capability for Suicide Scale; INQ, Interpersonal Needs Questionnaire; IUS‐12, Intolerance of Uncertainty Scale‐Short; TFII, Three Factor Impulsivity Scale.

*p < .05.

**p < .01.

***p < .001.

### Aim 1: effect of ERI factors and IU on interpersonal‐psychological theory risk factors

#### Capability for suicide

We next considered the effects of ERI factors and IU on the interpersonal‐psychological theory risk factors. Despite the lack of significant correlation between capability for suicide and either ERI or IU, we nevertheless examined the simultaneous regression per our a priori analytic plans. Results of this model (overall model *F* (3, 165) = 3.12, *p* = .028, adjusted *R* ^*2*^ = .036) showed that after controlling for both ERI factors and IU, higher levels of Feelings Trigger Action and lower levels of IU were independently correlated with capability for suicide (see Table 3). There was no significant effect of Pervasive Influence of Feelings on capability for suicide.

**TABLE 3:** Linear regression results for model predicting capability for suicide (ACSS).

Variable	*B*	*SE B*	*β*	*t*	*p*
(Constant)	34.54	3.90		8.86	<.001
Feelings Trigger Action (TFII)	**3.20**	**1.43**	.**21**	**2.23**	.**027**
Pervasive Influence of Feelings (TFII)	0.46	1.36	.04	0.34	.737
Intolerance of Uncertainty (IUS‐12)	**−0.28**	**0.13**	**−.22**	**−2.22**	.**028**

*Note*: Measures used for each variable are added in parentheticals for additional clarity.

Abbreviations: ACSS, Acquired Capability for Suicide Scale; IUS‐12, Intolerance of Uncertainty Scale‐Short; TFII, Three Factor Impulsivity Scale.

#### Exploratory follow‐up analyses with ACSS‐FAD and ACSS no‐FAD

Given concerns about the psychometrics of the full 20‐item version of the ACSS (see Section 2 above), we conducted exploratory follow‐up analyses replacing the full 20‐item ACSS with the ACSS‐FAD and with the ACSS no‐FAD (i.e, the 13‐item version of the ACSS omitting the seven ACSS‐FAD items).

Full results are presented in Table 4 below. The model utilizing the ACSS‐FAD (overall model *F* (3, 165) = 1.82, *p* = .146, adjusted *R* ^*2*^ = .01) showed that after controlling for both ERI factors and IU, lower levels of IU were independently correlated with the capability for suicide FAD items, but there was no significant effect of Feelings Trigger Action or Pervasive Influence of Feelings on the capability for suicide FAD items. In the model utilizing ACSS no‐FAD (overall model *F* (3, 165) = 7.34, *p* < .001, adjusted *R* ^*2*^ = .10), the pattern of results was identical to the model utilizing the full 20‐item ACSS: higher levels of Feelings Trigger Action and lower levels of IU were independently correlated with the capability for suicide, and there was no significant effect of Pervasive Influence of Feelings. Note that the adjusted *R* ^*2*^ of the model utilizing ACSS no‐FAD items was higher (0.10) than the adjusted *R* ^*2*^ of the model utilizing the full 20‐item ACSS (.036).

**TABLE 4:** Linear regression results for capability for suicide models using ACSS‐FAD and ACSS no‐FAD.

Variable	ACSS‐FAD (7 items)					ACSS no‐FAD (13 items)				
	*B*	*SE B*	*β*	*t*	*p*	*B*	*SE B*	*β*	*t*	*p*
(Constant)	16.13	2.12		7.61	<.001	16.42	2.12		7.76	<.001
Feelings Trigger Action (TFII)	0.14	0.77	.02	0.18	.860	**4.10**	**0.73**	.**45**	**5.60**	**<.001**
Pervasive Influence of Feelings (TFII)	0.50	0.74	.07	0.67	.502	0.66	0.73	.08	0.91	.366
Intolerance of Uncertainty (IUS‐12)	**−0.16**	**0.06**	**−.23**	**−2.51**	.**013**	**−0.21**	**0.06**	**−.26**	**−3.39**	**<.001**

*Note*: Measures used for each variable are added in parentheticals for additional clarity.

Abbreviations: ACSS, Acquired Capability for Suicide Scale; IUS‐12, Intolerance of Uncertainty Scale‐Short; TFII, Three Factor Impulsivity Scale.

#### Thwarted belongingness

We then considered the effects of ERI factors and IU on thwarted belongingness (see Table 5). Only Pervasive Influence of Feelings was a significant correlate of thwarted belongingness, such that greater Pervasive Influence of Feelings related to greater thwarted belongingness (overall model *F* (3, 165) = 11.57, *p* < .001, adjusted *R* ^*2*^ = .30).

**TABLE 5:** Linear regression results for model predicting thwarted belongingness (INQ).

Variable	*B*	*SE B*	*β*	*t*	*p*
(Constant)	4.76	3.29		1.45	. 150
Feelings Trigger Action (TFII)	1.61	1.21	.11	1.33	.185
Pervasive Influence of Feelings (TFII)	**5.13**	**1.15**	.**42**	**4.45**	**<.001**
Intolerance of Uncertainty (IUS‐12)	.14	0.11	.11	1.30	.194

*Note*: Measures used for each variable are added in parentheticals for additional clarity.

Abbreviations: INQ, Interpersonal Needs Questionnaire; IUS‐12, Intolerance of Uncertainty Scale‐Short; TFII, Three‐Factor Impulsivity Scale.

#### Perceived burdensomeness

Then, we examined the effects of ERI factors and IU on perceived burdensomeness (see Table 6). Both Feelings Trigger Action and Pervasive Influence of Feelings were significantly related to greater perceived burdensomeness, such that higher levels of each ERI factor related to higher perceived burdensomeness (overall model *F* (3, 165) = 27.63, *p* < .001, adjusted *R* ^*2*^ = .32).

**TABLE 6:** Linear regression results for model predicting perceived burdensomeness (INQ).

Variable	*B*	*SE B*	*β*	*t*	*p*
(Constant)	−3.61	2.24		−1.61	.109
Feelings Trigger Action (TFII)	**3.20**	**0.82**	.**30**	**3.89**	**<.001**
Pervasive Influence of Feelings (TFII)	**2.89**	**0.78**	.**34**	**3.69**	**<.001**
Intolerance of Uncertainty (IUS‐12)	.01	0.07	.01	0.13	.898

*Note*: Measures used for each variable are added in parentheticals for additional clarity.

Abbreviations: INQ, Interpersonal Needs Questionnaire; IUS‐12, Intolerance of Uncertainty Scale‐Short; TFII, Three‐Factor Impulsivity Scale.

### Aim 2: testing the interactivity of ERI and IU on interpersonal–psychological theory risk factors

For each of the three interpersonal‐psychological theory factors, we examined the effects of the interaction of IU x Feelings Trigger Action and IU x Pervasive Influence of Feelings, using hierarchical linear regression models to test these effects while controlling for the direct effects shown in Tables 3, 4, 5, 6. None of the interaction terms were significant in any of the models (all Beta's < .04, *p*'s > .059), suggesting no significant synergistic effect between ERI and IU.

## DISCUSSION

In the current study, we sought to clarify the role of ERI and IU in relation to risk factors from the interpersonal‐psychological theory of suicide. One novel facet of this study was that we differentiated between two factors of ERI: Pervasive Influence of Feelings, covering more internal cognitive and motivational over‐reactivity to emotion, as compared to Feelings Trigger Action, covering more behavioural over‐reactivity to emotion. A second novel facet was our test of IU as a direct correlate of the interpersonal‐psychological theory factors, and as an amplifier of the effects of ERI on those risk factors. We begin by discussing the profile of findings for ERI, and then we turn to the findings for IU.

Whereas previous work highlights the differential effects of these two forms of ERI on suicidal ideation vs. behaviour, no work had considered how this might extend to the interpersonal‐psychological theory. In line with our hypotheses, these two forms of ERI showed differential relations with risk factors for suicidal ideation vs. behaviour. Greater Feelings Trigger Action, but not Pervasive Influence of Feelings, was associated with greater capability for suicide. We also found partial support for our hypotheses that perceived burdensomeness and thwarted belongingness would be related to Pervasive Influence of Feelings but not Feelings Trigger Action: thwarted belongingness was only significantly associated with Pervasive Influence of Feelings, but perceived burdensomeness was significantly and positively associated with both Pervasive Influence of Feelings and Feelings Trigger Action.

Our finding that Feelings Trigger Action but not Pervasive Influence of Feelings was correlated with greater capability for suicide coheres with existing empirical evidence. Past literature suggests that it is the *type* of impulsive response, rather than general impulsivity itself, that leads an individual to develop capability for suicide (Anestis et al., 2014). Critically, we only found evidence for ties between Feelings Trigger Action and capability for suicide when we created a version of the Acquired Capability for Suicide Scale (ACSS; Van Orden et al., 2008) that dropped items assessing fear of death and thus only included items assessing pain tolerance and reactions to painful and provocative experiences (we called this the ACSS no‐FAD). In contrast, when analysing a version of the ACSS that *only* included items assessing fear about death (the ACCS‐FAD; Ribeiro et al., 2014), we found no significant relationship with Feelings Trigger Action. This nuanced finding suggests that behavioural ERI (i.e. Feelings Trigger Action) is more strongly associated with people's pain tolerance and their reactions to painful and provocative events than their level of fear about death. It may be that individuals higher in Feelings Trigger Action are prone to respond with impulsive behaviour in response to heightened emotions, and thus may be more likely to impulsively engage in risky and provocative behaviours, or painful behaviours like non‐suicidal self‐injury. In line with this, past work has demonstrated an association between greater Feelings Trigger Action and risky sex, reckless driving (Casini et al., 2020), and substance use (Johnson et al., 2017). In contrast to Feelings Trigger Action, the Pervasive Influence of Feelings ERI factor indexes reflexive thoughts, but not actions. The distinct relationships suggested by our findings have important implications for clinicians and for future research. Clinicians might consider explicitly distinguishing between a person's behavioural and cognitive impulsivity in response to emotion when attempting to understand their risk for suicide, and should also seek to understand how ERI may differentially relate to fear about death versus pain tolerance and reactions to painful and provocative experiences. We caveat that our analyses distinguishing between the ACSS‐FAD and the ACSS no‐FAD were exploratory and should be replicated in future work and perhaps with other measures of painful and provocative experiences, given there are concerns about the psychometrics of the ACSS (Ribeiro et al., 2014). However, if our findings hold in other studies and samples, future studies might test a proposed mediation path wherein higher Feelings Trigger Action increases suicidal behaviour via its effect on pain tolerance and reactions to painful and provocative events.

We found that thwarted belongingness was significantly associated with Pervasive Influence of Feelings, but not with Feelings Trigger Action, supporting our hypotheses. The significant relationship between thwarted belongingness and Pervasive Influence of Feelings may be explained by their shared ties with rumination. Both thwarted belongingness (Lin et al., 2022) and Pervasive Influence of Feelings (Johnson et al., 2022) show strong associations with rumination. Indeed, in recent work, the effect of Pervasive Influence of Feelings on suicidal ideation was not significant when considered conjointly with rumination (Johnson et al., 2022). While not directly tested in this study, it is possible that high Pervasive Influence of Feelings may influence thwarted belongingness through its links with rumination, but past research has not tested this within a mediation framework. Future work should further investigate rumination as one means through which Pervasive Influence of Feelings may increase thwarted belongingness.

Although we had hypothesized that perceived burdensomeness would only be significantly associated with Pervasive Influence of Feelings, we found that both Pervasive Influence of Feelings and Feelings Trigger Action were significantly associated with perceived burdensomeness. One tentative explanation for the Feelings Trigger Action effect could be that those high on this trait engage in risky sexual, driving, and aggressive behaviours (Casini et al., 2020; Johnson et al., 2017) that negatively impact their friends and family, which could create a sense of burden and strife. Clearly, more work is needed on the processes linking Pervasive Influence of Feelings and Feelings Trigger Action with the interpersonal‐psychological theory factors of perceived burdensomeness and thwarted belongingness; that work would ideally integrate other related risk variables, such as rumination.

A second novel facet of this work was to integrate IU as a direct risk factor for the interpersonal‐psychological theory factors, and as an amplifier of the effects of ERI. We found no evidence for interactions of ERI and IU, and the main effect of IU on capability for suicide was in the opposite direction than hypothesized: greater IU was significantly associated with lower capability for suicide. While this inverse finding is surprising and requires further empirical investigation, we tentatively propose that this result could be attributable to the relationship between intolerance of uncertainty and risk perception. Bredemeier and Berenbaum (2008) found that people higher in IU are more likely to overestimate the probability and costs of future negative outcomes. Individuals with higher IU may be less likely to engage in the types of actions that would cultivate the ability for suicide (nonsuicidal self‐injury, military combat, physical fights, thrill‐seeking activities) due to increased fear of negative outcomes. Future work should more rigorously test how IU relates to capability for suicide, and should specifically probe how fear of negative outcomes influences this relationship. Additionally, given our study utilized a cross‐sectional sample, it cannot speak to the causality or directionality of this effect. IU is thought of as a more dispositional trait underlying the development of later psychopathology (Carleton, 2016a), and research has found prospective relationships between temperament and IU, such that children with more neurotic temperament at age 3 show higher IU at age 12–15 (Hawes et al., 2021). This may suggest IU as a more basic and early‐developing trait that could precede any changes in an individual's capability for suicide, and future longitudinal work should probe for causality and directionality in the relationship between IU and capability for suicide. Should future work support the link between IU and capability for suicide, it would also be important to closely consider clinical implications of this effect. Because higher IU is prospectively linked with worse anxiety and mood disorder symptoms (Shapiro et al., 2020) and with increased suicidal ideation (Allan et al., 2021; Zerach & Levi‐Belz, 2019), IU should not be considered a protective factor against suicide in general. However, it may play a constrained role in specifically lowering people's ability to engage in the dangerous or harmful behaviour that connotes capability for suicide. It may be useful for clinicians to distinguish between low and high IU individuals and assess how their relationship to uncertainty relates to their capability for suicide.

Finally, we did not find evidence for links between IU and thwarted belongingness or perceived burdensomeness, nor did we find support for our hypotheses about interactivity between ERI factors and IU. Overall, with the exception of the effect of IU on capability for suicide (discussed above), our results suggest that IU does not influence the cognitive constructs of the interpersonal‐psychological theory, nor does IU act interactively with ERI to shape suicide risk. As with all null effects, it is difficult to understand exactly why we did not find significant relationships between IU, ERI, and perceived burdensomeness and thwarted belongingness. One potential reason for the null effect of IU on perceived burdensomeness and thwarted belongingness could be due to the strong effects of ERI factors on these outcomes. Although IU relates to perceived burdensomeness and thwarted belongingness at the basic association level, those effects become non‐significant after controlling for ERI factors within the same model. ERI appears to be a more potent predictor of these outcomes, supporting previous research on the strong link between ERI, suicidal ideation, and nonsuicidal self‐injury (Johnson et al., 2022). Further research is needed to better understand whether IU may uniquely predict cognitive risk factors for suicide above and beyond the contributions of other predictors, such as ERI. Overall, these results suggest that IU may not be the most potent treatment target to consider for reducing cognitive risk factors for suicide. Clinical interventions focused on reducing ERI, not IU, may be more effective at lowering thwarted belongingness and perceived burdensomeness.

Our findings should be considered in light of several limitations. First, we recruited participants via Amazon MTurk. MTurk has been extensively used to investigate psychological metrics and has been demonstrated to be more representative of the general population than typical convenience sampling (Arditte et al., 2016). However, there are concerns about the quality of data obtained via the platform (Thomas & Clifford, 2017). To bolster against this concern, our analyses excluded participants who completed the survey in an implausibly short amount of time or who showed evidence of careless responding (Meade & Craig, 2012), including failing at least one attention check and/or indicating the same response for every item in a scale. Relatedly, the generalizability of our findings may be limited by the fact that our sample was primarily white and drawn from a non‐clinical population. That said, we attempted to boost the clinical relevance of our results by screening for and only recruiting individuals with a lifetime history of suicidal ideation. An additional limitation is that the cross‐sectional nature of our study does not establish causality of any observed associations. Furthermore, there is a need for research examining more momentary temporal data to better understand acute predictors of suicidal behaviours (Chu et al., 2017; Franklin et al., 2017). Future work using ecological momentary assessment could shed light on how ERI ties to in‐the‐moment suicidal thoughts and behaviour. Finally, in our focus on the more proximal cognitive‐behavioural predictors of perceived burdensomeness, thwarted belongingness, and capability for suicide, our investigation did not test how the interpersonal‐psychological construct of hopelessness may relate to ERI or IU. Within the context of the fully elaborated version of the interpersonal‐psychological theory of suicide, wherein hopelessness about perceived burdensomeness and thwarted belongingness gives rise to suicidal desire (Joiner, 2005), it would be interesting to consider the potential roles of ERI and IU. For example, perhaps greater ERI in the presence of high levels of hopelessness connotes greater risk for greater suicidal desire.

## CONCLUSION

Our findings provide compelling evidence regarding the nuanced relationship between ERI and the components of the interpersonal‐psychological theory. Our results that both Pervasive Influence of Feelings and Feelings Trigger Action positively correlated with interpersonal‐psychological theory factors also suggest that intervening upon these ERI factors may prove fruitful in reducing downstream risk for suicide. This is especially pertinent given recent evidence that ERI can be successfully reduced using a brief online intervention and that intervening on ERI can reduce nonsuicidal self‐injury symptoms at 3‐month follow‐up (Johnson et al., 2020). In addition, our findings suggest an interesting association between higher IU and lower capability for suicidal behaviour. It is our hope that these findings spur research replicating and extending our results. Should our results be replicated, we believe they can help guide the development and implementation of more effective early suicidal interventions.

## AUTHOR CONTRIBUTIONS

**Amelia S. Dev:** Data curation; investigation; writing – original draft. **Theresa Davison:** Formal analysis; writing – original draft. **Hannah C. Broos:** Conceptualization; writing – original draft. **Sheri L. Johnson:** Conceptualization; funding acquisition; supervision; writing – review and editing. **Kiara R. Timpano:** Conceptualization; funding acquisition; project administration; supervision; writing – review and editing.

## CONFLICT OF INTEREST STATEMENT

The authors declare no conflicts of interest.

## Data Availability

The data that support the findings of this study are available on request from the corresponding author. The data are not publicly available due to privacy or ethical restrictions.
